# Comparative Respiratory Physiology in Cetaceans

**DOI:** 10.3389/fphys.2020.00142

**Published:** 2020-03-03

**Authors:** Andreas Fahlman, Alicia Borque-Espinosa, Federico Facchin, Diana Ferrero Fernandez, Paola Muñoz Caballero, Martin Haulena, Julie Rocho-Levine

**Affiliations:** ^1^Research Department, Fundación Oceanogràfic de la Comunitat Valenciana, Valencia, Spain; ^2^Global Diving Research Inc., Ottawa, ON, Canada; ^3^University of Valencia, Valencia, Spain; ^4^Biology Department, Avanqua Oceanogràfic SL, Valencia, Spain; ^5^Vancouver Aquarium, Vancouver, BC, Canada; ^6^Dolphin Quest Oahu, Honolulu, HI, United States

**Keywords:** diving physiology, marine mammals, bottlenose dolphin, killer whale, beluga, pilot whale, harbor porpoise, gray whale

## Abstract

In the current study, we used breath-by-breath respirometry to evaluate respiratory physiology under voluntary control in a male beluga calf [*Delphinapterus leucas*, body mass range (*M*_b_): 151–175 kg], an adult female (estimated *M*_b_ = 500–550 kg) and a juvenile male (*M*_b_ = 279 kg) false killer whale (*Pseudorca crassidens*) housed in managed care. Our results suggest that the measured breathing frequency (*f*_R_) is lower, while tidal volume (*V*_T_) is significantly greater as compared with allometric predictions from terrestrial mammals. Including previously published data from adult bottlenose dolphin (*Tursiops truncatus*) beluga, harbor porpoise (*Phocoena phocoena*), killer whale (*Orcinus orca*), pilot whale (*Globicephala scammoni*), and gray whale (*Eschrichtius robustus*) show that the allometric mass-exponents for *V*_T_ and *f*_R_ are similar to that for terrestrial mammals (*V*_T_: 1.00, *f*_R_: −0.20). In addition, our results suggest an allometric relationship for respiratory flow ($\dot{V}$), with a mass-exponent between 0.63 and 0.70, and where the expiratory $\dot{V}$ was an average 30% higher as compared with inspiratory $\dot{V}$. These data provide enhanced understanding of the respiratory physiology of cetaceans and are useful to provide proxies of lung function to better understand lung health or physiological limitations.

## Introduction

Comparing respiratory traits between terrestrial and marine mammals shows some striking differences in that when normalized by body mass, breathing frequency (*f*_R_) is generally lower and tidal volume (*V*_T_) greater in marine mammals (Kooyman, 1973; Piscitelli et al., 2013; Fahlman et al., 2017). Respiratory flow ($\dot{V}$) is also generally greater in marine mammals as compared with terrestrial species, especially in cetaceans, that have been shown to be able to generate expiratory flows ($\dot{V}$_exp_) that are at least one order of magnitude greater than in humans (Olsen et al., 1969a; Kooyman et al., 1971, 1975; Kooyman and Cornell, 1981; Piscitelli et al., 2013; Fahlman et al., 2015, 2017, 2019b). There appears to be great variability in the mechanical properties of the respiratory system, but in general marine mammals appear to have more compliant lung parenchyma as compared with terrestrial species, and a rib cage that allows the alveoli to compress and collapse without apparent trauma (Olsen et al., 1969b; Leith, 1976, 1989; Fahlman et al., 2011, 2017, 2018b; Denk et al., 2020). However, the limited availability of data on respiratory physiology has been, until recently, mostly limited to pinnipeds with very few estimates in cetaceans (Piscitelli et al., 2013; Fahlman et al., 2017). Data from different species are therefore useful to help determine allometric differences within and between marine species, and in comparisons with terrestrial mammals.

The aim of this study was to provide new comparative estimates of respiratory function from the false killer whale (*Pseudorca crassidens*), and the beluga (*Delphinapterus leucas*) while breathing at the surface at rest. In addition, we performed an allometric analysis of available lung function data from the bottlenose dolphin (*Tursiops truncatus*) (Fahlman et al., 2019a, d), beluga (Kasting et al., 1989; Fahlman et al., 2019b), pilot whale (*Globicephala scammoni*, now called *Globicephala macrorhynchus*) (Olsen et al., 1969b), harbor porpoise (*Phocoena phocoena*) (Reed et al., 2000), killer whale (*Orcinus orca*) (Kasting et al., 1989), and gray whale (*Eschrichtius robustus*) (Wahrenbrock et al., 1974; Kooyman et al., 1975). Our data and analysis provide results that confirm that *f*_R_ is generally lower and *V*_T_ is greater as compared with terrestrial mammals, and the allometric mass-exponent for $\dot{V}$, *V*_T_, and *f*_R_ is similar to terrestrial mammals. In addition, while *V*_T_ is greater as compared with similar sized terrestrial mammals, it is seldom close to vital or total lung capacity (Fahlman et al., 2020), as has often been assumed (Dolphin, 1987). Finally, the allometric relationship for $\dot{V}$ provides interesting opportunities to estimate lung function by recording the respiratory flow noise, a method called phonspirometry in the human literature and currently being tested in dolphins (Sumich, 2001; van der Hoop et al., 2014). With proxies that allow remote recording of *f*_R_ and *V*_T_, improved estimates of field metabolic rate (FMR) may be possible in cetaceans (Fahlman et al., 2016). Thus, this improved knowledge of respiratory physiology in cetaceans is not only important to enhance basic knowledge in comparative respiratory physiology, but a better understanding of normal respiratory capacity and limitations will also have important implications to aid conservation of charismatic megafauna. We therefore analyze published and unpublished lung function data from 7 cetacean species of varying size (body mass).

## Materials and Methods

### Animals

Breath-by-breath respirometry was used to measure $\dot{V}$ while staying calm at the side of a pool from: one adult female and one juvenile male false killer whale housed at Sea Life Park (Hawaii- United States, January 2018) and Vancouver Aquarium (Vancouver-Canada, September 2016), respectively, and one male beluga calf at the Oceanogràfic (Valencia-Spain, April-June 2016) (Table 1). In addition, we included previously published respiratory data from 11 adult male and 3 adult female Atlantic bottlenose dolphins (Fahlman et al., 2019a, d), 6 adult and 2 juvenile beluga (Kasting et al., 1989; Fahlman et al., 2019b), 1 pilot whale (Olsen et al., 1969b), 2 harbor porpoises (Reed et al., 2000), 4 killer whales (Kasting et al., 1989), and 2 gray whales (Wahrenbrock et al., 1974; Kooyman et al., 1975). The animal identification (ID), sex, body mass (*M*_b_), and year of birth (known or estimated) are summarized in Table 1. For the female false killer whale, the *M*_b_ was estimated from length and girth, while for all other animals *M*_b_ was measured. The study protocols were accepted at each facility, as well as by the Animal Care and Welfare Committee at the Oceanogràfic (OCE-17-16, amendments OCE-29-18 and OCE-3-19i), and the Bureau of Medicine (BUMED, NRD-1015).

**TABLE 1:** Animal identification (ID), species false killer whale (*Pseudorca crassidens-Pc)*, beluga (*Delphinapterus leucas –Dl*), Atlantic bottlenose dolphin (*Tursiops truncatus-Tt*), harbor porpoise (*Phocoena phocoena-Pp*), pilot whale (*Globicephala scammoni-Gs*), killer whale (*Orcinus orca*-*Oo*), gray whale (*Eschrichtius robustus-Er*), number of animals (N), sex (F-female, M-male, number behind abbreviation is number of animals), body mass (*M*_b_, kg), and approximate year of birth or age for wild caught animals or year of birth for animals born under human care.

**Animal ID**	**Species**	**N**	**Sex**	**M_b_ (kg)**	**Birth year/estimate age (Yr)**	**References**
Pc1	Pc	1	F	500–545	30+	–
Pc2	Pc	1	M	279	2014	–
Tt	Tt	14	F3/M11	140–235	1989–2013	Fahlman et al., 2019a
Dl1	Dl	1	M	160 ± 15 (162 ± 20)	2016	–
Dl2	Dl	5	F3/M2	450–891	1986–2007	Fahlman et al., 2019b
Dl3	Dl	3	F2/M1	385–620	Adult1/Juvenile2	Kasting et al., 1989
Gs	Gs	1	NA	450	Adult	Olsen et al., 1969b
Er1	Er	1	NA	1116–1745 (4.77–5.78)!	Calf	Kooyman et al., 1975
Er2	Er	1	F	2000–6000	Calf	Wahrenbrock et al., 1974
Pp	Pp	2	M2	28	Juvenile	Reed et al., 2000
Oo1	Oo	1	F	1090	NA	Spencer et al., 1967
Oo2	Oo	3	F1/M2	1650–3600	Adult1/Juvenile2	Kasting et al., 1989

*All data with a reference are previously published. ! Only length reported and *M*_b_ estimated from equation in Trites and Pauly (1998).*

### Experimental Trials

All experiments were performed using operant conditioning as previously detailed (Fahlman et al., 2019a, d). Participation by each individual was voluntary, and the animals were not restrained and could refuse to participate or withdraw at any point during the experimental trial. Each experiment (trial) consisted of an animal staying stationary in the water with the blow-hole out of the water, allowing the pneumotachometer to be placed over the blow-hole.

### Respiratory Flow

The procedures and equipment were identical to those used in our previous studies (Fahlman et al., 2015, 2018a,b, 2019a,b), and the procedure is briefly summarized here. The $\dot{V}$ was measured using a custom-made Fleisch type pneumotachometer (Micah Brodsky, V.M.D. Consulting, Miami, FL, United States; Mellow Design, Valencia, Spain), which housed a low-resistance laminar flow matrix (Item # Z9A887-2, Merriam Process Technologies, Cleveland, OH, United States). A differential pressure transducer (Spirometer Pod, ML 311, ADInstruments, Colorado Springs, CO, United States) was connected to the pneumotachometer with two firm walled, flexible tubes (310 cm lengths of 2 mm I.D.). The differential pressure transducer was connected to a data acquisition system (Powerlab 8/35, ADInstruments, Colorado Springs, CO, United States), and the data was captured at 400 Hz and displayed on a laptop computer running LabChart (v. 8.1, ADInstruments, Colorado Springs, CO, United States). A low resistance diffuser was added to homogenize the flow (Fahlman et al., 2018b), which helped resolve the difference in calibration factors for inspired (${\dot{V}}_{i\,n\,s\,p}$) and expired (${\dot{V}}_{\text{exp}}$) flow (Fahlman et al., 2015). To assess the flow range over which the flow was linear, we used an industrial fan (Atmosphere Vortex 728 CFM S Line S-800 Fan, 8″) to generate laminar flow up to 120 l⋅s^–1^, as measured by a calibrated industrial flow meter (Merriam Process Technologies, Serial No. Z50MC2-4-LHL, Flow standard serial No. WMMH10-6). The pneumotachometer was placed in series with the Merriam flow meter and the flow calibrated from 0 to 120 l⋅s^–1^, showing that the response was linear and identical in both directions. For each trial, the differential pressure was used to estimate$\dot{V}$, and was calibrated using a 7.0 l calibration syringe (Series 4900, Hans-Rudolph Inc., Shawnee, KS, United States). The signal was integrated and the *V*_T_ determined as detailed in a previous study (Fahlman et al., 2015). A normal breath was considered a respiration that began with an exhalation followed by an immediate inspiration.

### Data Assessment and Statistical Analysis

We compared the resting data within and between individuals and species. The relationship between a dependent variable (*V*_T_, *f*_R_, $\dot{V}$, and breath durations) and *M*_b_ was analyzed using linear-mixed effects models (lme, R: A Language and Environment for Statistical Computing, R Foundation for Statistical Computing, version 3.3.3, 2016). We log_10_-transformed the variables to generate linear functions that could be used with the lme function in R. Species was treated as a random effect, which accounted for the correlation between multiple measurements of the same species (Littell et al., 1998). Normality was confirmed using the qqnorm plot. Best models of remaining variables were chosen by the log-likelihood (LL) ratio test. Acceptance of significance was set to the *P* < 0.05 level, while 0.05 < *P* < 0.1 was considered a trend. Data are presented as the mean ± standard deviation, unless otherwise stated.

## Results

For the beluga calf, the average (±s.d., *n* = 78) *V*_T_, $\dot{V}$_*insp*_, $\dot{V}$_exp_, and *f*_R_ were, respectively, 4.0 ± 2.3 l, 7.4 ± 1.9 l s^–1^, 8.3 ± 1.4 l s^–1^, 1.9 ± 1.5 breaths min^–1^. The average *V*_T_, $\dot{V}$_*insp*_, $\dot{V}$_exp_, and *f*_R_ for the juvenile false killer whale (*n* = 7) were 16.9 ± 1.6 l, 38.6 ± 3.6 l s^–1^, 72.8 ± 7.5 l s^–1^, and 2.5 ± 2.4 breaths ⋅ min^–1^, and the same values for the adult false killer whale (*n* = 54) were, respectively, 13.5 ± 4.5 l, 32.8 ± 8.5 l s^–1^, 50.1 ± 18.0 l s^–1^, and 5.4 ± 3.3 breaths ⋅ min^–1^.

**FIGURE 1:**
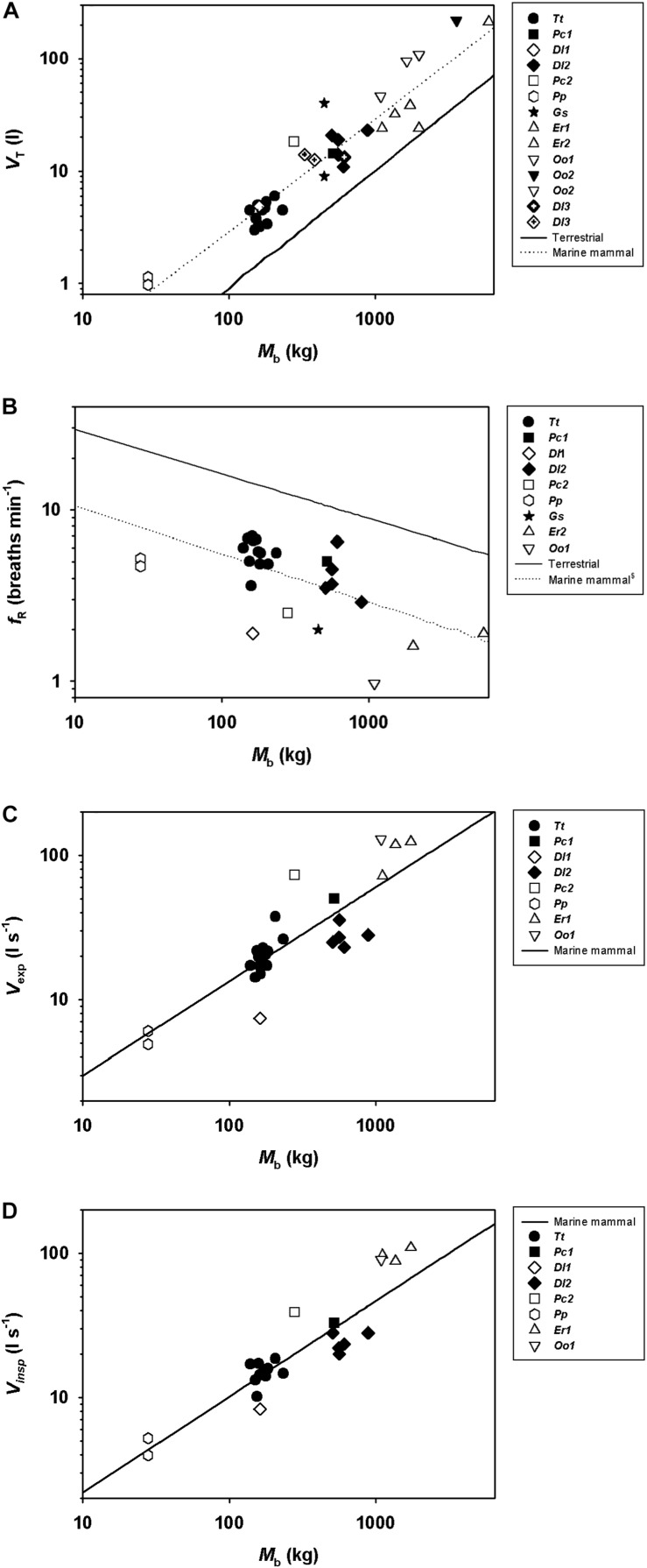
Scatter plots showing **(A)** tidal volume (*V*_T_), **(B)** breathing frequency (*f*_R_), **(C)** expiratory ($\dot{V}$_exp_), and **(D)** inspiratory flow ($\dot{V}$_insp_) during rest in adult bottlenose dolphins (*Tt, Tursiops truncatus*), a calf (*Dl1*, *Delphinapterus leucas*) and adult belugas (*Dl2*), a juvenile and an adult false killer whale (*Pc1* and *Pc2*, *Pseudorca crassidens*), adult pilot whale (*Gs*, *Globicephala scammoni*), juvenile harbor porpoises (*Pp*, *Phocoena phocoena*), adult and juvenile killer whale (*Oo1 and Oo2*, *Orcinus orca*), and calf gray whales (*Er1 and Er2, Eschrichtius robustus*). Solid line is for allometric predictions published for terrestrial mammals at rest (Stahl, 1967), and dotted line is prediction equation in Table 2.

**FIGURE 2:**
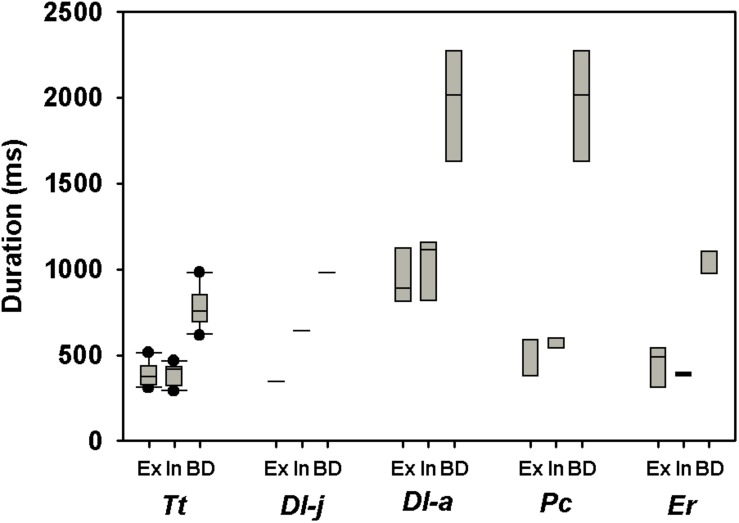
Box plot showing expired (Ex), inspired (In), and total (BD) breath-duration during rest in adult bottlenose dolphins (*Tursiops truncatus, Tt*), a beluga calf (*Delphinapterus leucas, Dl-j*) or adult (*Dl-a*), adult false killer whale (*Pseudorca crassidens, Pc*), and gray whale calves (*Eschrichtius robustus, Er*).

Including all available data from adult whales (Figure 1, the figure also includes data from juveniles and calves), and log_10_-transforming *M*_b_ (log[*M*_b_]), *V*_T_ (log[*V*_T_]), $\dot{V}$ (log[$\dot{V}$]), and *f*_R_ [log(*f*_R_)], there was a significant relationship between log(*M*_b_) and log(*V*_T_), log(*f*_R_), expired log($\dot{V}$), and inspired log($\dot{V}$) (Table 2). Neither, expiratory (χ^2^ = 2.33, 1 df, *P* < 0.1), inspiratory (χ^2^ = 1.49, 1 df, *P* < 0.01) or total breath duration (χ^2^ = 2.30, 1 df, *P* < 0.1, Figure 2) changed with *M*_b_.

**TABLE 2:** Results from linear mixed model for tidal volume (*V*_T_), breathing frequency (*f*_R_), and respiratory flow $\left(\dot{V}\right)$.

**Dependent variable**	**β_0_**	**log[*M*_b_]**	**χ^2^**	***P*-value**
log[*V*_T_]	−1.50 ± 0.23	1.00 ± 0.08	67.2	<0.01
log[*f*_R_]	0.97 ± 0.28	−0.20 ± 0.10	13.7	<0.00
Log[${\dot{V}}_{\text{exp}}$]	−0.23 ± 0.21	0.70 ± 0.12	36.2	<0.01
Log[${\dot{V}}_{\text{insp}}$]	−0.17 ± 0.09	0.63 ± 0.10	43.2	<0.01

*The models included all available data for adult cetaceans only (Figures 1, 2), and to account for allometric changes and to adjust for heteroscedasticity the data were log_10_-transformed, e.g. *M*_*b*_ (log[*M*_*b*_]), *V*_*T*_ (log[*V*_*T*_]), *f*_*R*_ [log(*f*_*R*_)], and *$\dot{V}$* (log[$\dot{V}$]).*

## Discussion

In the current study we provide new respiratory measurements from spontaneous breaths that were within the range of those that provide accurate flow estimates (Finucane et al., 1972; Fahlman et al., 2019b), on a male beluga calf, a male juvenile and an adult female false killer whale. We also provide an allometric analysis of previously published data from adult male and female cetaceans. Our results suggest that the predicted *f*_R_ is lower, while *V*_T_ is significantly greater as compared with terrestrial mammals (Figures 1A,B). The reported data also suggest an allometric relationship for $\dot{V}$ with a mass-exponent between 0.65 and 0.66, and where $\dot{V}$_exp_ is an average 30% higher as compared with $\dot{V}$_insp_ in the *M*_b_ range of the reported data (Figures 1C,D).

In the current study our analysis provided estimated allometric relationships between *V*_T_, *f*_R_, $\dot{V}$, and *M*_b_ (Figure 1 and Table 2). When predicting allometric relationships for metabolism there are standard conditions to assure that confounding variables are controlled. For basal metabolic rate (BMR), animals are to be resting adults in a post-absorptive state, measured under thermoneutral conditions. However, no definition such as basal *V*_T_ or *f*_R_ currently exist, and our measurements were not done in fasted animals and not all were adults. While it is known that digestion significantly increase metabolic rate (Secor, 2009), we are not aware of any study that assess variation in respiratory variables following feeding (Crosfill and Widdicombe, 1961; Stahl, 1967; Lasiewski and Calder, 1971; Schroter, 1980; Calder, 1981; Bennett and Tenney, 1982; Feldman, 1995). However, our measurements provide comparative results in juvenile and adult cetaceans at rest. Thus, these data provide valuable comparisons between species and with terrestrial mammals. For example, the mass-exponent for *V*_T_ for adult cetaceans reported in the current study (cetacean: 1.00) scale similarly, with those reported in both terrestrial and marine mammals (marine mammal: 0.97; terrestrial: 1.04) (Stahl, 1967; Bishop, 1997; Fahlman et al., 2019c) but the gain appears to be different, possibly reflecting the different challenges with an aquatic life.

There are clear differences in respiratory physiology between terrestrial and marine species, with the latter having lower *f*_R_ and greater *V*_T_ (Figures 1A,C; Stahl, 1967; Mortola and Limoges, 2006; Fahlman et al., 2017). While the relative *V*_T_ is greater in marine mammals (19–22 ml kg^–1^, this study and Mortola and Sequin, 2009; Fahlman et al., 2017, 2019c) as compared with terrestrial mammals (8 ml⋅kg^–1^) (Stahl, 1967), the allometric mass-exponent were similar between marine and terrestrial mammals. Thus, respiratory function scales similarly, between these groups of mammals, possibly reflecting similar metabolic demands. In the current study, the allometric mass-exponent between *f*_R_ and *M*_b_ (−0.20 ± 0.10) was similar to that reported for terrestrial mammals (−0.26, Stahl, 1967), but lower than that reported previously in marine mammals (−0.42, Mortola and Sequin, 2009). One possible reason for this difference is that placement of the flow meter over the blow-hole requires desensitization to avoid altering the behavior. Without proper desensitization individual animals may not have had enough time to get used to the procedure resulting in periods of hyperventilation. For example, several dolphins included in the current study began a trial with periods of high *f*_R_ with shallow *V*_T_. Through repeated training we have observed that these animals become used to the measurements and calm down, progressing to a more physiological breathing pattern. As an example, one beluga in our previous study (S5, Fahlman et al., 2019b) and the adult false killer whale had unexpectedly high *f*_R_ (5.4 breaths ⋅ min^–1^ vs. 2.7 breaths ⋅ min^–1^ from Table 2), and were also individuals where we had limited ability to extend the training and desensitization. Conversely, the juvenile beluga had much lower *f*_R_ than that predicted from the allometric equation (1.9 breaths ⋅ min^–1^ vs. 3.4 breaths ⋅ min^–1^). Thus, the relatively higher and lower *f*_R_ in these individuals may have increased the variation. For the previous study with a lower mass-exponent, on the other hand, the *f*_R_ obtained were by focal observations of 19 species of marine mammals (Mortola and Limoges, 2006), which may have prevented changes due to placement of the pneumotachometer over the blow-hole. It is also possible that some breaths are missed during focal observations, which may also explain the differences between studies.

In terrestrial mammals, both total lung capacity and *V*_T_ scale isometrically with *M*_b_, while *f*_R_ scales allometrically (Stahl, 1967), resulting in a minute ventilation that scales similar to metabolic rate with a mass-exponent around −0.25. In marine mammals, the reported mass-exponent for *V*_T_ is 0.97 (Fahlman et al., 2020), which agrees with the results from the current study, and for *f*_R_ between −0.20 (this study) and −0.42 (Mortola and Limoges, 2006). Thus, we would therefore expect the mass-exponent for minute ventilation, the product between *f*_R_ and *V*_T_, to range between −0.60 and −0.80. Using the former, the mass-corrected minute ventilation for the false killer whales would be 523 ml ⋅ kg^–0.80^ ⋅ min^–1^ and 552 ml ⋅ kg^–0.80^ ⋅ min^–1^ for the juvenile and adult false killer whales, respectively. Thus, these animals achieved similar minute ventilations using different ventilatory strategies, where the juvenile animal had a 17% lower *f*_R_ and a 92% higher *V*_T_, while the adult had a 102% higher *f*_R_ and an 18% lower *V*_T_ as compared with the allometric equation. The mass-corrected minute ventilation for the beluga calf, on the other hand, was 131 ml ⋅ kg^–0.80^ ⋅ min^–1^. Thus, the beluga calf had a 44% lower *f*_R_ and 21% lower *V*_T_ as compared with the allometric results presented in this study, possibly indicating that this young animal was performing surface breath-holds. While at first these results appear inconsistent, they agree with the literature that both *f*_R_ and *V*_T_ vary considerably, but in general the former is significantly lower while the latter is considerably greater as compared with terrestrial mammals (Mortola and Limoges, 2006; Piscitelli et al., 2013; Fahlman et al., 2017, 2020). On-going development of bio-logging tags may allow measurement of *f*_*H*_ (Cauture et al., 2019) or respiratory flow noise (e.g. phonospirometry; Sumich and May, 2009; van der Hoop et al., 2014) that could provide estimates of *f*_R_ and *V*_T_ in undisturbed adult marine mammals during rest, which could help verify the current and past estimates.

Phonospirometry is a method that uses the respiratory sound to estimate $\dot{V}$, and uses the flow noise and the assumption that increasing sound level scales linearly with$\dot{V}$. Phonospirometry has previously been used successfully to estimate *V*_T_ and respiratory health in humans, and recent validation work that links to flow noise to $\dot{V}$ has been performed in dolphins (van der Hoop et al., 2014). The data in the current study provide a useful linking function between flow noise and $\dot{V}$ as we present evidence of a size dependent change in $\dot{V}$ that scales with a mass-exponent that is similar for inspired and expired breaths (Table 2). Thus, phonospirometry validation studies in smaller species, such as dolphins, belugas or pilot whales, would allow us to scale $\dot{V}$ to larger species, such as sperm whales or humpback whales, and provide a method to more reliably estimate *V*_T_. Improved estimates of *V*_T_ and how it changes during different activities would significantly help to improve our understanding of the physiological responses of these animals. In addition, a better estimate of how *V*_T_ changes during or following different activities, such as active swimming or diving, would allow improved estimates of field metabolic rates in studies using *f*_R_ to estimate energy use (Fahlman et al., 2016).

This is the first study to report comparative resting respiratory function ($\dot{V}$, *V*_T_, *f*_R_, breath duration) in small and medium sized cetaceans. Our data suggest that while the intercepts are different, the allometric mass-exponents for *V*_T_ and *f*_R_ in cetaceans are similar to terrestrial mammals. The relationship in $\dot{V}$ and animal size may provide improved estimates of lung function and field metabolic rates in large species. The data presented in the current study provide valuable comparisons within and between species that are important to understand physiological limitations in cetaceans. Furthermore, these data highlight the significance of access to animals under managed care that provide physiological measurements under voluntary conditions.

## Data Availability Statement

The data used in this study are freely available upon request to afahlman@whoi.edu.

## Ethics Statement

The study protocols were accepted at each facility, as well as by the Animal Care and Welfare Committee at the Oceanogràfic (OCE-17-16, amendments OCE-29-18 and OCE-3-19i), and the Bureau of Medicine (BUMED, NRD-1015).

## Author Contributions

AF conceived the project, obtained animal care permits, developed the analytical approach, and wrote the manuscript. AF, JR-L, MH, PM, DF, and AB-E carried out fieldwork. AF, AB-E, and FF completed data and statistical analyses. All authors contributed to the editing of the manuscript.

## Conflict of Interest

AF was associated with Global Diving Research Inc. during the time of the study. The remaining authors declare that the research was conducted in the absence of any commercial or financial relationships that could be construed as a potential conflict of interest.

## Abbreviations

$\dot{V}$: respiratory flow
*f*_R_: breathing frequency
s*f*_R_: mass-specific breathing frequency
*sV*_T_: mass-specific tidal volume
*V*_T_: tidal volume.
